# Predictors of Successful First-Line *Helicobacter pylori* Eradication with Fluoroquinolones in Pakistan: A Prospective Exploration of Demographic and Clinical Factors

**DOI:** 10.3390/antibiotics13030211

**Published:** 2024-02-23

**Authors:** Sumaira Khadim, Iyad Naeem Muhammad, Tanveer Alam, Shahnaz Usman, Hina Rehman, Sajjad Haider

**Affiliations:** 1 Faculty of Health Sciences, Iqra University of Health Sciences, Karachi 75500, Pakistan; 2 Faculty of Pharmacy & Pharmaceutical Sciences, University of Karachi, Karachi 75270, Pakistan; iyadnaeem@uok.edu.pk (I.N.M.); haider.sajjadd@gmail.com (S.H.); 3 Jamal Noor Hospital, Karachi 74800, Pakistan; alamtanviralam@yahoo.com; 4 RAK College of Pharmacy, RAK Medical & Health Sciences University, Ras Al Khaimah 11172, United Arab Emirates; shahnaz.usman@rakmhsu.ac.ae; 5 Institute of Pharmaceutical Sciences, Jinnah Sindh Medical University, Karachi 75510, Pakistan; hina.rehman@jsmu.edu.pk

**Keywords:** *H. pylori*, demographic variables, clinical variables, eradication failure, moxifloxacin, levofloxacin, RCT

## Abstract

Growing antibiotic resistance complicates *H. pylori* eradication, posing a public health challenge. Inconclusive research on sociodemographic and clinical factors emphasizes the necessity for further investigations. Hence, this study aims to evaluate the correlation between demographic and clinical factors and the success rates of *H. pylori* eradication. A group of 162 *H. pylori*-positive patients were allocated randomly to receive either a ten-day moxifloxacin-based triple therapy or a levofloxacin-based sequential therapy. Eradication success was determined through the stool antigen test. Logistic regression analysis was utilized to figure out potential factors that contribute to *H. pylori* eradication success. Significantly higher *H. pylori* eradication rates were observed in the middle age group (COR: 3.671, *p* = 0.007), among females (*p* = 0.035), those with BMI ≥ 25 (COR: 2.011, *p* = 0.045), and non-smokers (COR: 2.718, *p* = 0.018). In multivariate analysis, age and smoking emerged as significant predictors (*p* < 0.05). Patients with comorbidities, excluding diabetes and hypertension (COR: 4.432, *p* = 0.019), dyspepsia (COR: 0.178, *p* < 0.001), and moxifloxacin triple therapy (COR: 0.194, *p* = 0.000), exhibited higher chances of eradication (*p* < 0.05). Further research is vital for tailored approaches to enhance eradication success.

## 1. Introduction

*Helicobacter pylori (H. pylori)* infection is widely identified as the primary causative agent of gastritis and has been significantly linked with the pathogenesis of duodenal and gastric ulcer disease, as well as gastric cancer [1]. Based on global estimations, it has been determined that prevalence of infection is approximately 44.3% (95%CI: 40.9–47.7%) in the general population. Notably, this infection exhibits a higher prevalence in developing nations [2]. The continent of Asia, characterized by a population surpassing 2 billion individuals, has shown a significant prevalence of *H. pylori* infection, contributing to approximately 75% of the global burden of stomach cancer cases [3]. It is crucial to acknowledge that the prevalence of *H. pylori* infection is exceptionally elevated in Pakistan, with rates ranging from 85% to 95% [4].

Despite ongoing efforts to enhance treatment strategies, the rates of *H. pylori* eradication continue to fall below the desired level of effectiveness [5]. Factors such as demographic and clinical characteristics, along with antibiotic resistance, are considered potential contributors to treatment failure in *H. pylori* eradication [6]. 

Numerous studies have examined the effectiveness of various eradication protocols and their potential association with demographic variables. Age, gender, ethnicity, occupation, socioeconomic status, smoking habits, and body mass index have all been identified as significant factors in *H. pylori* eradication, yet outcomes exhibit notable global variation [7,8,9,10,11,12,13,14]. 

Moreover, it is imperative to underscore the profound significance of clinical characteristics among patients in the context of *H. pylori* eradication. A multitude of research investigations have elucidated an increased susceptibility to eradication failure in patients with certain clinical conditions, notably including diabetes [15], hypertension [16], the presence of endoscopic lesions [17], and the manifestation of dyspepsia [18].

Hence, the successful eradication of *H. pylori* infection is contingent upon a multifaceted interplay of social, environmental, genetic, and clinical factors. Therefore, a critical need exists for conducting region-specific inquiries to comprehensively elucidate a thorough evaluation of the risks and benefits, as well as tailor the treatment approach to each individual, in order to effectively eliminate *H. pylori* infection in patients. Therefore, the goal of this research was crafted to assess the demographic and clinical factors in relation to *H. pylori* eradication success rates.

## 2. Results

### 2.1. Characteristics of the Study Participant 

Table 1 presents the baseline demographic and clinical features of the study participants. A total of 162 *H. pylori*-positive patients (84 males, 78 females), exhibiting a mean age of 51 years (range: 18–85 years), were included. The majority of participants were married (72%), and 55% reported a low income level, with no reported family history of H. pylori infection (85%). Upon initial enrollment, the symptom that was most commonly reported by patients was abdominal discomfort, which accounted for 63.5% (103 out of 162) of the cases. This was followed by dyspepsia, reported by 55.5% (90 out of 162) of the patients, and gastroesophageal reflux, reported by 48.1% (78 out of 162) of the patients. Following the endoscopic evaluation, gastritis was found to be the most commonly observed condition, with a prevalence of 70.3% (114 out of 162 cases), significantly surpassing the occurrence of other identified findings. In terms of treatment regimens, 52% of patients received a moxifloxacin-based triple therapy, while 47.5% underwent a levofloxacin-based sequential therapy.

### 2.2. Univariate Analysis of Demographic Factors influencing H. pylori Eradication 

Table 2 displays the outcomes of the univariate analysis examining the impact of demographic factors on *H. pylori* eradication. The findings reveal a significantly higher eradication rate in the middle age group (30–49 years; *p*-value 0.001), among females compared to males (*p*-value 0.035), in patients with a higher BMI (≥25) (*p*-value 0.044), and in individuals who have never smoked (*p*-value 0.015). No significant effects were observed regarding income level, marital status, or family history in relation to *H. pylori* eradication.

### 2.3. Logistic Regression Analysis of Demographic Factors Influencing H. pylori Eradication 

Table 3 presents the logistic regression analyses analyzing the impact of demographic factors on eradication therapy. Variables that showed significant associations in the univariate analysis were included in the bivariate and multivariate analyses. In the bivariate logistic regression analysis, three variables—age, BMI, and smoking—emerged as significant factors influencing eradication therapy. Patients within the middle age group (30–39 years) had 3.6 times higher chances of eradication compared to both the younger and older age groups (COR: 3.671; 95%CI (1.432–9.416); *p* = 0.007). Individuals with a BMI ≥25 demonstrated 2 times higher chances of eradication than those with a BMI < 25 (COR: 2.011; 95%CI (1.014–3.987); *p* = 0.045). Non-smokers had 2.7 times higher chances of eradication compared to current smokers (COR: 2.718; 95%CI (1.190–6.208); *p* = 0.018). In the multivariate analysis, where adjustments were made for all variables, only age and smoking remained significant predictors for eradication (*p*-value < 0.05).

### 2.4. Univariate Analysis of Clinical Factors influencing H. pylori Eradication 

Table 4 presents the outcomes of the univariate analysis investigating the influence of clinical factors on eradication of *H. pylori* infection. The results demonstrate a notably higher eradication rate in patients with comorbid conditions other than diabetes or hypertension (*p*-value 0.012), among patients experiencing dyspepsia and abdominal pain (*p*-values 0.001 and 0.048, respectively), and in those treated with moxifloxacin-based therapy compared to levofloxacin sequential therapy (*p*-value 0.002). However, no significant effects were observed concerning lesion characteristics, gastroesophageal reflux, premature fullness, weight loss, and loss of appetite in relation to *H. pylori* eradication.

### 2.5. Logistic Regression Analysis of Clinical Factors Influencing H. pylori Eradication 

Table 5 displays the outcomes of both bivariate and multivariate logistic regression analyses investigating the impact of clinical factors on eradication therapy. Variables that demonstrated significant associations in the univariate analysis were incorporated in both the bivariate and multivariate analyses.

In the bivariate logistic regression analysis, four variables—comorbidities, dyspepsia, abdominal pain, and treatment group—emerged as significant factors influencing eradication therapy. Patients with comorbidities other than diabetes and hypertension had 4.4 times higher chances of eradication compared to those with diabetes or hypertension (COR: 4.432; 95%CI (1.272–15.436); *p* = 0.019). Individuals with dyspepsia exhibited 5.6 times higher chances of eradication (COR: 0.178; 95%CI (0.085–0.377); *p* < 0.001). Patients with abdominal pain had 1.99 times higher chances of eradication (COR: 0.502; 95%CI (0.252–1.001); *p* = 0.05). Those treated with moxifloxacin triple therapy had 5 times higher chances of eradication compared to those treated with levofloxacin-based sequential therapy (COR: 0.194; 95%CI (0.090-0.419); *p* = 0.000). In the multivariate analysis, where adjustments were made for all variables, comorbidities, dyspepsia, and treatment group remained significant predictors of eradication (*p*-value less than 0.05).

## 3. Discussion

This study strongly confirms that the failure of *H. pylori* eradication is closely connected to various demographic and clinical factors. The findings suggest a notable increase in failure among both young and elderly individuals, while the middle-aged group exhibited higher eradication rates. These results align with the existing literature. The study conducted by Yokota et al. revealed a noteworthy correlation between the middle-aged group and successful eradication therapy, even after accounting for various other factors, in comparison to other age groups [19]. Middle-aged individuals’ higher success in *H. pylori* eradication may be linked to increased motivation, possibly driven by receiving therapy recommendations from health checkups. This group showed greater adherence to medication, suggesting a more conscientious approach to treatment compared to older patients [19]. This finding is also similar to prior studies that identified “forgetting to take the drug” as a contributing factor to treatment failure in young and elderly patients [20]. Moreover, age has been found to be a factor influencing *H. pylori* eradication failure in both developed and developing countries. In developed countries, patients aged 45 years and above have been associated with an increased risk of eradication failure [21]. Conversely, in developing countries, there is no notable distinction in the eradication of *H. pylori* between younger and older age groups [22]. These findings suggest that the impact of age on *H. pylori* eradication outcomes may vary across different socioeconomic and healthcare contexts, emphasizing the importance of considering regional factors when addressing treatment strategies for this infection.

Smoking appeared as a significant factor negatively impacting the eradication of *H. pylori*. Multiple studies consistently demonstrate that smoking plays a pivotal role, showing a contributing factor to the failure of eradicating *H. pylori* infection, especially among males [14,23,24]. This observation aligns with the findings of recent research. Smoking may increase gastric acid production, reducing blood flow and mucus secretion. This could hinder the transport of antibiotics to the gastric mucosa, compromising their effectiveness in *H. pylori* eradication [25,26].

In addition, smoking has been found to increase the failure rate of *H. pylori* eradication treatment in both developed and developing countries. In a meta-analysis conducted in a developing country, it was observed that smoking increases the failure rate of *H. pylori* eradication treatment, and the risk of failure is higher with a current smoking status and a high smoking dose, while the use of vonoprazan appears to mitigate this effect [27]. Another study conducted in Japan found that smoking did not exacerbate *H. pylori* eradication failure when using vonoprazan as compared to proton pump inhibitors (PPIs) [28]. Therefore, smoking can negatively impact the efficacy of *H. pylori* eradication therapy in both developed and developing countries if not carefully considered in the selection of treatment protocols and medications.

Moreover, the current study identified a statistically significant association between the existence of diabetes or hypertension and the eradication of *H. pylori* even after adjusting for other factors. These findings align with the outcomes reported in the meta-analysis undertaken by Song et al., encompassing both developed and developing countries. The meta-analysis indicated a notably heightened risk of eradication failure in individuals with type 2 diabetes mellitus (T2DM) (odds ratio = 2.59 [95% confidence interval, 1.82–3.70], *p* < 0.001) for *H. pylori* infection [29]. In study conducted by Kato et al. in Japan, participants with diabetes had a 3.7% eradication failure rate, slightly higher than the 2.5% rate in those without diabetes, but the difference lacked statistical significance (1.2%, 95% CI −0.8% to 3.2%) [30]. Regarding hypertension, this study aligns with Byun et al.’s investigation reporting a 66.7% *H. pylori* eradication rate among hypertensive patients, though statistical significance was not reached in the study [16]. The suboptimal eradication rate in type 2 diabetes may result from disruptions in drug pharmacokinetics [31], increased susceptibility to infections [29], and compromised immune function [32]. 

Additionally, this study highlights a robust and statistically significant connection between *H. pylori* elimination and the relief of dyspeptic symptoms. In multivariate logistic regression, dyspepsia remains noteworthy with an odds ratio of 0.218 (*p*-value of 0.000), even after adjusting for other variables. These findings align with Fiorini et al.’s research on a 12-day triple-treatment regimen using rifabutin, showing an 89.9% *H. pylori* eradication rate among those with dyspepsia [18]. In contrast, a prospective trial conducted by Hulst et al. revealed that the eradication rates for peptic ulcer disease were higher (73%) in comparison to non-ulcer dyspepsia (55%), indicating a significant distinction (*p*-value = 0.016) [33]. The ongoing controversy around the eradication of *H. pylori* in non-ulcer dyspepsia prompts an exploration of *H. pylori* strains [34], revealing a higher prevalence of dyspeptic symptoms with CagA-positive strains in functional dyspepsia patients [35]. These divergent results underscore the complexity of the relationship, necessitating further investigation into contributing variables.

This investigation further elucidates the influence of a moxifloxacin-based triple therapy on the eradication rate of *H. pylori*. The efficacy of treatment outcomes is notably influenced by two pivotal factors: suboptimal patient adherence stemming from intricate treatment regimens and the escalating incidence of antibiotic-resistant infections [36]. The existing literature indicates a rising resistance in levofloxacin-based regimens, and the intricacy of these regimens exacerbates challenges in patient compliance [37]. Consequently, moxifloxacin emerges as a more favorable option for *H. pylori* eradication therapy, offering potential advantages over levofloxacin in mitigating resistance concerns and addressing issues related to treatment adherence [37].

Moreover, moxifloxacin-based therapy has shown higher efficacy in *H. pylori* eradication in both developed and developing countries. In a study conducted in Iraq, the utilization of the moxifloxacin triple regimen for *H. pylori* eradication demonstrates similar efficacy levels comparable to those of the quadruple regimen, surpassing the eradication rates achieved with the clarithromycin triple regimen. Furthermore, moxifloxacin triple therapy exhibits greater tolerability and does not contribute to an increased incidence of overall adverse effects when contrasted with other regimens employed in this study [38]. Similarly, another study in South Korea reported a higher eradication rate with moxifloxacin-based triple therapy compared to clarithromycin-based triple therapy [39].

This study uniquely explores a broad range of *H. pylori* eradication risk factors, enhancing the likelihood of discovering associations often missed in more narrowly focused studies. However, its focus on demographic and clinical factors may limit a comprehensive understanding, excluding variables like genetics and environment. 

Since this investigation was confined to a specific center, the characteristics of the participants, the healthcare practices, and the overall environment may be unique to that particular setting. This uniqueness poses challenges when attempting to extrapolate the results to a more diverse or broader population. The external validity of the findings—meaning the extent to which they can be generalized beyond the immediate study setting—may be compromised.

Additionally, a single-center study may not adequately capture the variability present in different demographics, geographic locations, or healthcare systems. Consequently, caution is warranted when attempting to apply the results to settings that differ significantly from the environment in which this study was conducted. While this research contributes valuable insights within the confines of our single center, it is essential to recognize the limitations associated with generalizability. In future research endeavors, consideration should be given to multi-center designs that encompass a more varied and representative sample of populations and settings, thereby enhancing the external validity of the study’s conclusions.

## 4. Materials and Methods

### 4.1. Patient Recruitment

The current study is a prospective, randomized clinical trial conducted at Jamal Noor Hospital in Karachi, Pakistan, within its Gastroenterology Department, which operates as a 100-bedded secondary care facility. It is an open-label, single-center investigation. During the time frame encompassing June 2020 to June 2022, adult individuals who fulfilled the diagnostic criteria for *H. pylori* infection through upper gastrointestinal system endoscopy or stool antigen testing employing the Rapid Strip HpSA Kit (Safe care Biotech, Hangzhou, China) and being aged over 18 years were recruited. A biopsy sample, acquired through endoscopy, was formalin-fixed and employed for assessing *H. pylori* infection through Giemsa staining. Positive outcomes in the rapid urease tests were determined when the gel exhibited a pink or red coloration at room temperature within 24 hours following the examination. 

### 4.2. Ethics

This study protocol received approval from the IRB of the University of Karachi, under letter No. IBC No. IBC KU-76/19, and was recorded as a randomized clinical trial according to standard ClinicalTrials.gov identifier NCT05863858. Before being enrolled, all participants in the study were required to provide written informed consent. The research conducted in this study followed the guidelines set forth by Good Clinical Practice standards and adhered to the rules outlined in the Declaration of Helsinki [40,41].

### 4.3. Randomization and Treatment 

Patients were randomly assigned to the MAO or LAO-LTO groups using a method based on a lottery draw. Identical slips, half marked “M” and half marked “L”, were folded, shuffled in a basket, and drawn by blindfolded participants. “M” slips corresponded to the MAO group, and “L” slips corresponded to the LAO-LTO group. The process was repeated until the desired sample size was achieved [42]. The participants were allocated to receive one of two treatment protocols for a duration of 10 consecutive days.

LAO-LTO group: levofloxacin 500 mg BID, amoxicillin 1 gm BID, and omeprazole 20 mg BID for first five days followed by levofloxacin 500 mg BID, tinidazole 500 mg BID, and omeprazole 20 mg BID.

MAO group, N = 70: moxifloxacin 400 mg OD, amoxicillin 1 gm BID, and omeprazole 20 mg BID.

### 4.4. Sample Size

The determination of the sample size was performed utilizing the Openepi WHO online calculator for sample size. With eradication rates of 91.3% and 71.6% in both groups, a confidence level of 95%, a 5% significance level, and test power of 80%, the computed minimum sample size was found to be 124, which could be equally divided into two groups with 62 participants in each [39]. Therefore, in each group, more than 75 patients were enlisted to ensure the attainment of more precise and reliable results.

### 4.5. Study Design

The resident pharmacists were provided with instructions to complete a questionnaire that encompassed inquiries pertaining to the demographic information, including age, gender, BMI, income level, marital status, smoking behaviors, familial history of Helicobacter Pylori, and clinical information including any co-existing medical conditions, endoscopic diagnosis, and pre-treatment symptoms, of the recruited participants. The influence of these demographic and clinical factors on *H. pylori* eradication was kept as the primary outcome of this study. The determination of the eradication rate was carried out by performing stool antigen testing between the 4th and 6th weeks following the treatment. The testing was performed by the microbiology laboratory using the Rapid Strip HpSA Kit. The technique employed was an enzyme immunoassay with a monoclonal antibody [43]. 

### 4.6. Statistical Analysis

Chi-square and Fisher’s exact test were utilized to investigate the association between demographic and clinical variables and their corresponding treatment groups by IBM-SPSS 26.0. *H. pylori* eradication rates were assessed, and the binary logistic regression model was employed to calculate crude odds ratios. Moreover, the adjusted odds ratio was determined to assess the magnitude of the association between *H. pylori* eradication and potential factors. The significance threshold was set at a *p*-value below or equal to 0.05.

## 5. Conclusions

In conclusion, the effective eradication of *H. pylori* is intricately linked to various demographic and clinical variables, such as age, smoking habits, selected treatment regimens, comorbidities, and the presence of dyspepsia. The impact of these factors underscores the need for personalized approaches to optimize success rates in treatment. As highlighted in this study, tailoring interventions based on a patient’s specific characteristics can significantly enhance the likelihood of eradication success and concurrently reduce the risk of associated complications. To refine and tailor these therapeutic strategies further, ongoing and future research is imperative. A comprehensive understanding of the interplay between these factors not only facilitates the development of more effective eradication protocols but also contributes to improved overall health outcomes for individuals grappling with *H. pylori* infections.

## Figures and Tables

**Table 1:** Demographic and clinical characteristics of patients.

Variables	N = 162	(%)
Demographic Characteristics		
Age		
<30	43	26.5
30–39	47	29
40–49	63	38.8
≥50	9	5.5
Gender		
Male	84	51.8
Female	78	48.1
BMI		
<25	65	40.1
≥25	97	59.8
Income Level in PKR		
≥51 K	25	15.4
25 K–50 K	48	29.6
<25 K	89	54.9
Marital Status		
Married	117	72.2
Unmarried	45	27.7
Smoking Status		
Current smoker	29	17.9
Never smoked	133	82
Family history of *H. Pylori*		
Yes	24	14.8
No	138	85.1
Clinical Characteristics		
Comorbidities		
Diabetes or hypertension	133	82
Others	29	17.9
Lesion Characteristics		
Gastritis	114	70.3
Gastric/duodenal ulcer	36	22.2
Reflux oesophagitis	12	7.4
Pre-Treatment Symptoms		
Dyspepsia	90	55.5
Abdominal pain	103	63.5
Gastroesophageal reflux	78	48.1
Premature fullness	73	45
Weight loss	15	9.2
Loss of appetite	41	25.3
Treatment Groups		
MAO	85	52.4
LAO-LTO	77	47.5

M: moxifloxacin; A: amoxicillin; O: omeprazole; L: levofloxacin; T: tinidazole.

**Table 2:** Univariate analysis of demographic factors influencing *H. pylori* eradication.

Demographic Variables	Success (*n* = 114)	Failure (*n* = 48)	Eradication Rate (%)	*p*-Value
Age (in years)				
<30	23	20	53.4	0.001 *
30–39	38	9	80.8	
40–49	50	13	79.3	
≥50	3	6	33.3	
Gender				
Male	53	31	63	0.035 *
Female	61	17	78	
BMI				
<25	40	25	61.5	0.044 *
≥25	74	23	76.2	
Income Level in PKR				
≥51 K	19	6	76	0.637
25 K–50 K	35	13	72.9	
<25 K	60	29	67.4	
Marital Status				
Married	84	33	71.7	0.567
Unmarried	30	15	66.6	
Smoking Status				
Current smoker	15	14	51.7	0.015 *
Never smoked	99	34	74.4	
Family History *H. pylori*				
Yes	19	5	79.2	0.345
No	95	43	68.8	

Eradication rate (%) = Success/Success + Failure; *: significant *p*-value at 5%.

**Table 3:** Logistic regression analysis of demographic factors influencing *H. pylori* eradication.

Logistic Regression Model	Independent Variable	Crude Odd Ratio (95% C.I)	*p*-Value	Adjusted Odd Ratio (95% C.I)	*p*-Value
Age	<30	1		1	
	30–39	3.671 (1.432–9.416)	0.007 *	4.519 (1.646–12.407)	0.003 *
	40–49	3.344 (1.422–7.866)	0.006 *	4.249 (1.700–10.621)	0.002 *
	≥50	0.435 (0.096–1.968)	0.280	0.353 (0.075–1.664)	0.188
Gender	Male	1		1	
	Female	1.424 (0.717–2.827)	0.313	1.999 (0.915–4.365)	0.082
BMI	<25	1		1	
	≥25	2.011 (1.014–3.987)	0.045 *	1.176 (0.252–5.491)	0.836
Smoking	Current Smoker	1		1	
	Never Smoked	2.718 (1.190–6.208)	0.018 *	3.436 (1.404–8.412)	0.007 *

*: significant *p*-value at 5%.

**Table 4:** Univariate analysis of clinical factors influencing *H. pylori* eradication.

Clinical Variables		Success (*n* = 114)	Failure (*n* = 48)	Eradication Rate (%)	*p*-Value
Comorbidities					
Diabetes or hypertension		88	45	66.1	0.012 *
Others		26	3	89.6	
Lesion Characteristics					
Gastritis		83	31	72.8	0.150
Gastric/duodenal ulcer		21	15	58.3	
Reflux esophagitis		10	2	83.3	
Pre-Treatment Symptoms					
Dyspepsia	Yes	77	13	85.6	0.001 *
	No	37	35	51.4	
Abdominal pain	Yes	78	25	75.7	0.048 *
	No	36	23	61	
Gastroesophageal Reflux	Yes	56	22	71.8	0.702
	No	58	26	69	
Premature Fullness	Yes	48	25	65.8	0.244
	No	66	23	74.2	
Weight Loss	Yes	8	7	53.3	0.129
	No	106	41	72.1	
Loss of Appetite	Yes	25	16	61	0.127
	No	89	32	73.6	
Treatment Groups					
MAO		69	16	81.2	0.002 *
LAO-LTO		45	32	58.4	

Eradication rate (%) = Success/Success + Failure; *: significant *p*-value at 5%.

**Table 5:** Logistic regression analysis of clinical factors influencing *H. Pylori* eradication.

Logistic Regression Model	Independent Variable	Crude Odd Ratio (95% C.I)	*p*-Value	Adjusted Odd Ratio (95% C.I)	*p*-Value
Comorbidities	Diabetes or hypertension	1		1	
	Others	4.432 (1.272–15.436)	0.019 *	5.831 (1.579–21.527)	0.008 *
Dyspepsia	Yes	1		1	
	No	0.178 (0.085–0.377)	<0.001 *	0.218 (0.098–0.482)	0.000 *
Abdominal Pain	Yes	1		1	
	No	0.502 (0.252–1.001)	0.05 *	0.773 (0.351–1.700)	0.521
Treatment Group	MAO	1		1	
	LAO-LTO	0.194 (0.090–0.419)	0.000 *	0.233 (0.103–0.525)	0.000 *

*: significant *p*-value at 5%.

## Data Availability

Data are contained within the article.
